# DELLA proteins modulate *Arabidopsis* defences induced in response to caterpillar herbivory

**DOI:** 10.1093/jxb/ert420

**Published:** 2014-01-07

**Authors:** Zhiyi Lan, Sebastian Krosse, Patrick Achard, Nicole M. van Dam, Jacqueline C. Bede

**Affiliations:** ^1^Department of Plant Science, McGill University, 21111 Lakeshore, Ste-Anne-de-Belleuve, QC, H9X 3V9, Canada; ^2^Ecogenomics, Radboud University Nijmegen, Heyendaalseweg 135, 6525 AJ Nijmegen, The Netherlands; ^3^Institut de Biologie Moléculare des Plantes, Université de Strasbourg, Strasbourg, France

**Keywords:** *Arabidopsis thaliana*, caterpillar labial saliva, DELLA proteins, gibberellins, induced plant defences, *Spodoptera exigua*.

## Abstract

Upon insect herbivory, many plant species change the direction of metabolic flux from growth into defence. Two key pathways modulating these processes are the gibberellin (GA)/DELLA pathway and the jasmonate pathway. In this study, the effect of caterpillar herbivory on plant-induced responses was compared between wild-type *Arabidopsis thaliana* (L.) Heynh. and quad-*della* mutants that have constitutively elevated GA responses. The labial saliva (LS) of caterpillars of the beet armyworm, *Spodoptera exigua*, is known to influence induced plant defence responses. To determine the role of this herbivore cue in determining metabolic shifts, plants were subject to herbivory by caterpillars with intact or impaired LS secretions. In both wild-type and quad-*della* plants, a jasmonate burst is an early response to caterpillar herbivory. Negative growth regulator DELLA proteins are required for the LS-mediated suppression of hormone levels. Jasmonate-dependent marker genes are induced in response to herbivory independently of LS, with the exception of *AtPDF1.2* that showed LS-dependent expression in the quad-*della* mutant. Early expression of the salicylic acid (SA)-marker gene, *AtPR1*, was not affected by herbivory which also reflected SA hormone levels; however, this gene showed LS-dependent expression in the quad-*della* mutant. DELLA proteins may positively regulate glucosinolate levels and suppress laccase-like multicopper oxidase activity in response to herbivory. The present results show a link between DELLA proteins and early, induced plant defences in response to insect herbivory; in particular, these proteins are necessary for caterpillar LS-associated attenuation of defence hormones.

## Introduction

Confronted with caterpillar attack, plants often redirect metabolic flux away from growth and into defensive compounds (Schwachtje and Baldwin, 2008). These physiological processes are regulated through distinct hormone-mediated pathways which shape the plant’s response. In general, jasmonic acid (JA) and related compounds are implicated in plant defence responses against chewing insect herbivores, whereas gibberellins (GAs) promote plant growth and development (Ballare, 2011; Erb *et al.*, 2012). In addition, caterpillar salivary effectors modulate plant defences, often suppressing JA-induced plant responses (Musser *et al.*, 2002; Bede *et al.*, 2006; Weech *et al.*, 2008; Diezel *et al.*, 2009; Tian *et al.*, 2012).

When *Arabidopsis thaliana* (L.) Heynh is wounded by caterpillar herbivory, a rapid, transient increase in jasmonate biosynthesis results in the accumulation of the bioactive form of JA, 7-jasmonoyl-*l*-isoleucine (JA-Ile) (Fonseca *et al.*, 2009). By bridging jasmonate ZIM-domain (JAZ) proteins with the E3 ubiquitin ligase SCF^COI1^ complex, JA-Ile promotes the targeted degradation of the JAZ protein by the 26*S* proteasome, releasing MYC2/3/4 transcription factors, leading to induced plant responses (Chini *et al.*, 2007; Thines *et al.*, 2007; Katsir *et al.*, 2008; Yan *et al.*, 2009; Sheard *et al.*, 2010; Fernandez-Calvo *et al.*, 2011; Erb *et al.*, 2012). *Lipoxygenase 2* (*AtLOX2*), *Plant Defensin 1.2* (*AtPDF1.2*), and *Vegetative Storage Protein 2* (*AtVSP2*) are well characterized markers of MYC-regulated gene expression (Bell and Mullet, 1993; Lorenzo *et al.*, 2004; Dombrecht *et al.*, 2007; Pre *et al.*, 2008; Robert-Seilaniantz *et al.*, 2011; Kazan and Manners, 2013), although late expression of *PDF1.2* is also positively regulated through TGA transcription factors (Zander *et al.*, 2010).

Activation of the jasmonate pathway results in the induction of the plant defence responses. In *Arabidopsis*, key defensive strategies include the production of antinutritive proteins, such as trypsin inhibitors (TIs) and laccase-like multicopper oxidase (LMCO), and secondary metabolites, such as glucosinolates (GSs) (Duffey and Stout, 1996; Van Poecke, 2007). In many plant systems, TIs are induced in response to caterpillar herbivory and bind to gut serine proteinases, impeding protein digestion and, hence, insect growth (Broadway *et al.*, 1986; van Dam *et al.*, 2001; Cipollini *et al.*, 2004; Clauss and Mitchell-Olds, 2004; Weech *et al.*, 2008; Tian *et al.*, 2012). LMCOs have diverse plant physiological functions, including interfering with protein digestion by oxidizing plant-derived polyphenolics in the insect gut, generating quinones that react with protein amino acid residues preventing their absorption (Zavala *et al.*, 2004; McCraig *et al.*, 2005; Constabel and Barbehenn, 2008). *Arabidopsis* and other members of the Brassicaceae also contain signature GSs (Brown *et al.*, 2003; Halkier and Gershenzon, 2006; Hopkins *et al.*, 2009). To date, ~200 GSs have been identified, which are broadly categorized into aliphatic, indole, and aromatic GSs (Clarke, 2010). Over 35 GSs have been identified in *Arabidopsis*, with representative GSs of the aliphatic and indoyl pathways, such as 3-hydroxylpropyl glucosinolate and glucobrassicin, respectively, being prominent in Landsberg (L*er*) leaves (Kliebenstein *et al.*, 2001; Brown *et al.*, 2003). Wounding by chewing insect herbivores disrupts cellular compartments, allowing contact between the enzyme myrosinase and vacuolar-localized GSs, generating a diversity of toxic and noxious compounds, such as (iso)thiocyanates and nitriles (Halkier and Gershenzon, 2006). The product that is formed and its toxicity to insect herbivores greatly depend on the GS side chain. Generalist caterpillars of the beet armyworm, *Spodoptera exigua* (Hübner), are adversely affected by the aliphatic class of GSs, whereas aphids are mainly affected by indole GSs (Li *et al.*, 2000; Wittstock *et al.*, 2003; Beekswilder *et al.*, 2004; Mewis *et al.*, 2005; Kusnierczyk *et al.*, 2007; Mosleh Arany *et al.*, 2008; van Dam and Oomen, 2008; Rohr *et al.*, 2012).

Caterpillar labial salivary (LS) effectors modulate the jasmonate pathway and subsequent induced defence responses. Usually, feeding damage as well as mechanical wounding increase the biosynthesis of jasmonate signalling hormones (Ballere, 2011). However, when responses are compared between plants fed upon by *S. exigua* caterpillars with intact or impaired LS secretions or when caterpillar LS is added to wounded plant tissues, these responses may be suppressed and/or delayed (Weech *et al.*, 2008; Diezel *et al.*, 2009; Tian *et al.*, 2012). Presently, evidence suggests that caterpillar LS-mediated suppression of induced plant defences involves the activation of the salicylic acid (SA)/nonexpressor of pathogenesis-related protein 1 (NPR1) pathway (Mur *et al.*, 2006; Weech *et al.*, 2008). *Spodoptera exigua* growth (biomass) was higher when caterpillars were fed on *coi1* mutant plants compared with *etr1* and *npr1* genotypes (Mewis *et al.*, 2005); this suggests that JA pathway COI1 is needed for defence responses but insects use the SA/NPR1 and ethylene pathways to circumvent plant defences, such as GSs. Noctuid caterpillar LS is rich in oxidoreductase enzymes, such as glucose oxidase (GOX), that is believed to be a key effector in the modulation of host plant responses (Eichenseer *et al.*, 1999; Musser *et al.*, 2002; Weech *et al.*, 2008; Afshar *et al.*, 2010; Eichenseer *et al.*, 2010). The hydrogen peroxide generated by GOX may act as an upstream signal activating the SA/NPR1 pathway (Shapiro and Zhang, 2001). Recently, Van der Does *et al.* (2013) showed that negative regulation of the JA-induced defences by the SA/NPR1 pathway occurs downstream of SCF^COI1^-mediated protein degradation instead through the ORA59 transcription factor. However, other plant hormone pathways, such as GAs, must also contribute to this cross-talk to optimize and fine-tune the plant’s response to changing environmental conditions.

Diterpenoid GA phytohormones promote growth-related physiological processes in flowering plants (Sun, 2011; Hauvermale *et al.*, 2012; Davière and Achard, 2013). Binding of GA to its receptor, Gibberellin Insensitive Dwarf 1 (GID1), leads to the degradation of the negative growth regulator DELLA proteins by the 26*S*–proteasome pathway (Sasaki *et al.*, 2003; Dill *et al.*, 2004; Fu *et al.*, 2004; Hartweck and Olsewski, 2006; Murase *et al.*, 2008; Shimada *et al.*, 2008). The five DELLA proteins in *Arabidopsis* exhibit temporal and spatial differences but are functionally redundant (Gallego-Bartolomé *et al.*, 2011; Hauvermale *et al.*, 2012). *Arabidopsis* quadruple-*della* (quad-*della*) mutant plants have knockouts in four of these five DELLA proteins, *gai-t6*, *rga-t2*, *rgl1-1*, and *rgl2-1*, resulting in constitutively elevated GA responses (Achard *et al.*, 2008).

Cross-talk between the GA and JA pathway most probably occurs via DELLA proteins (Hou *et al.*, 2010; Wild *et al.*, 2012; Yang *et al.*, 2012). In vegetative tissues, JA signalling induces expression of the gene encoding the DELLA protein RGL3 which competes with MYC2 for binding to JAZ proteins (Hou *et al.*, 2010; Wild *et al.*, 2012). Thereby, DELLA proteins act to enhance JA-induced defence responses by repressing the activity of negative regulator JAZ proteins. Also, by interfering with GA degradation of DELLA proteins, JA prioritizes defensive over growth-related pathways (Heinrich *et al.*, 2012; Yang *et al.*, 2012). In floral tissues, DELLA proteins interact directly with MYC2 to repress JA-dependent expression of genes encoding sesquiterpene synthases (Hong *et al.*, 2012). Since caterpillar LS-mediated suppression of induced plant defences is believed to involve effectors that generate reactive oxygen species (ROS), such as hydrogen peroxide, and DELLA proteins act to scavenge and reduce ROS levels, DELLA proteins may also play a role in plant–insect interactions by weakening caterpillar LS-dependent induced responses (Musser *et al.*, 2002; Bede *et al.*, 2006; Achard *et al.*, 2008; Weech *et al.*, 2008; Paudel *et al.*, 2013). Expression of *NPR1* is induced by treatment of *Arabidopsis* with GAs (Alonso-Ramírez *et al.*, 2009). This implies that DELLA proteins may act to suppress the NPR1 pathway that would, again, weaken caterpillar LS-mediated attenuation of induced responses.

In this study, *Arabidopsis* responses to herbivory by fourth instar *S. exigua* caterpillars were compared in wild-type Landsberg *erecta* (L*er*) and quad-*della* mutant plants. The role of LS in these interactions was determined by using caterpillars manipulated to generate two populations: one with intact LS secretions and the other with impaired LS secretions. The focus of this study was early changes at the hormonal, gene expression, and defensive protein and metabolite levels within the first 10h after the onset of herbivory to evaluate the role of JA versus GA trade-offs in this plant–insect interaction. Systemic changes in five plant hormones were recorded, including JA, its biologically active conjugate JA-Ile, and its precursor 12-oxo-phytodienoic acid (OPDA), which is also an important signalling molecule in plant–insect interactions (Farmer *et al.*, 2003; Taki *et al.*, 2005; Fonseca *et al.*, 2009). Additionally, changes in SA and abscisic acid (ABA) were analysed. Increases in ABA levels are often observed in response to mechanical wounding, possibly as a response to water loses due to the damage (Erb *et al.*, 2012). In addition, representative genes of the JA/ethylene pathway (*AtPDF1.2*), the JA/MYC2 pathway (*AtLOX2* and *VSP2*), and the SA pathway (*AtPR1*) were analysed. Expression of *AtPDF1.2b* (At2g26020) is negatively regulated by MYC2 (Penninckx *et al.*, 1998; Boter *et al.*, 2004; Lorenzo *et al.*, 2004; Dombrecht *et al.*, 2007; Pre *et al.*, 2008). In addition, late expression of this gene is further activated by the NPR1/TGA pathway (Zander *et al.*, 2010). LOX2 is the rate-limiting enzyme in JA biosynthesis and rapidly induced in response to jasmonate, wounding, or caterpillar herbivory (Bell and Mullet, 1993). *AtVSP2* expression is another marker for the MYC2 branch of the JA pathway (Dombrecht *et al.*, 2007). *Pathogenesis-related 1* (*AtPR1*, At2g14610) expression, a marker of the SA/NPR1 pathway, is induced in response to infection by biotrophic pathogens and aphids (Zhang *et al.*, 1999; Glazebrook, 2005; Mur *et al.*, 2006; Kusnierczyk *et al.*, 2007; Walling, 2008). Given the competition between DELLA proteins and MYC2 for the JAZ proteins, a decrease in positively regulated MYC2-dependent markers was expected in the quad-*della* mutant following insect herbivory (Hou *et al.*, 2010; Wild *et al.*, 2012; Wild and Achard, 2013). Also, since caterpillar LS effector(s) may exert the suppression of JA-induced responses through the generation of ROS, DELLA proteins scavenge these compounds, and DELLA proteins suppress the NPR1 pathway, a stronger caterpillar LS-dependent suppression of JA-mediated responses was expected in the quad-*della* mutants (Musser *et al.*, 2002; Bede *et al.*, 2006; Achard *et al.*, 2008; Weech *et al.*, 2008; Alonso-Ramírez *et al.*, 2009; Paudel *et al.*, 2013). In addition to measuring hormone levels and gene expression, other inducible plant defences, namely TI, LMCOs, and GS, that, alone or in combination, may negatively affect the herbivore, were also assessed.

## Materials and methods

### Chemicals

Chemicals used in this study were obtained from Sigma Chemical Company, unless otherwise specified.

### Plant cultivation

Wild-type *A. thaliana* cv L*er* and the quadr-*della* mutant (*gai-t6, rga-t2, rgl1-1, rgl2-1*) seeds were grown in pasteurized (80 °C for 2h) Agro Mix. After stratification at 4 °C for 2 d, the seeds germinated in a phytorium (8:16h light:dark, 250 μE m^–2^ s^–1^, 23 °C). As GAs regulate multiple aspects of plant development, wild-type and quad-*della* mutants were grown under short-day conditions to synchronize vegetative growth and prevent the onset of bolting and flowering (Cheng *et al.*, 2004; Davière and Achard, 2013). Plants were bottom watered as needed with dilute 0.15g l^–1^ N-P-K fertilizer. At ~2 weeks, plants were removed to leave three evenly spaced *Arabidopsis* plants per pot.

### Insect maintenance

*Spodoptera exigua* caterpillars were maintained on a meridic wheat germ-based artificial diet (Bio-Serv) (16:8h light:dark, 28–40% humidity, 22 °C). Eggs collected from mated adults were used to maintain the colony for >30 generations.

### Herbivory experiment

Plants ~5 weeks old [growth stages 1.11–1.14 (Boyes *et al.*, 2001)] were either kept as controls control (no insects) or subject to herbivory by fourth instar *S. exigua* caterpillars with intact (cat) or impaired (caut) LS secretions. To prevent LS secretions, caterpillar spinnerets were cauterized (caut insects) (Musser *et al.*, 2002). As caterpillar LS contains high levels of the enzyme GOX, success of cauterization was tested by allowing caterpillars to feed on glass discs pre-soaked in glucose/sucrose solution (5mg each sugar) and observing GOX activity through the peroxidase/3,3′-diaminobenzidine assay (Weech *et al.*, 2008). Both subsets of caterpillars (cat and caut) were allowed to feed on wild-type *Arabidopsis* for 12h before the beginning of the herbivory experiment to allow them to adjust to a plant diet.

To either the wild type (L*er*) or the quad-*della* mutant, three fourth instar caterpillars were placed in each pot that was then enclosed by netting to prevent caterpillar escape. As *S. exigua* caterpillars feed more actively at night, the experiments were initiated in the dark. Insects were placed on the plants 4h after the plant’s transition to dark. To minimize the effect of plant volatile signalling in the growth cabinets, pexiglass plates separated the different treatments (control, cat, caut).

After 10h, caterpillars were removed and plants were flash-frozen in liquid nitrogen. The three plants in each pot were pooled to prepare one sample. For hormone analysis, the entire above-ground portions of the plant were taken. For gene expression and defensive compound and protein analyses, only caterpillar-damaged leaves were collected to focus on local responses. Samples were stored in an –80 °C freezer until analysis. This experiment was repeated eight independent times. For hormone analysis, gene expression, and GS analysis, four biological replicates were analysed. For defensive protein and biomass loss experiments, eight biological replicates were used.

To calculate biomass loss, aerial tissues were dried for 3 d at 70 °C. From 20% to 29% of plant tissue was consumed by caterpillars, regardless of plant genotype. Cauterization of the caterpillar spinneret did not affect feeding.

### Hormone analysis

Lyophilized plant samples were ground using a TissueLyser (Qiagen) and tissues were sent to the Danforth Plant Science Centre for hormone analysis by liquid chromatography–mass spectroscopy/tandem mass spectroscopy (LC-MS/MS). Samples were spiked with deuterium-labelled internal standards of SA (D5-SA), ABA (D6-ABA), and JA (D2-JA). Samples were extracted in ice-cold methanol:acetonitrile (MeOH:ACN, 1:1, v/v) using a TissueLyser for 2min at a frequency of 15 Hz s^–1^ then centrifuged at 16 000 *g* for 5min at 4 °C. Supernatants were transferred to new tubes and the pellets re-extracted. After the supernatants were pooled, samples were evaporated using a Labconco Speedvac. Pellets were redissolved in 200 μl of 30% MeOH and analysed by LC-MS/MS.

LC separation was conducted on a Shimadzu system by reverse-phase chromatography on a monolithic C_18_ column (Onyx, 4.6 mm×100mm, Phenomenex). A gradient of 40% solvent A [0.1% acetic acid in HPLC-grade water (v/v)] held for 2min to 100% solvent B [90% ACN with 0.1% acetic acid (v/v)] for 5min was used with a flow rate of 1ml min^–1^. The LC system was interfaced with an AB Sciex QTRAP mass spectrometer equipped with a TurboIonSpray (TIS) electrospray ion source in negative mode. Parameters were set to: capillary voltage –4500, nebulizer gas (N_2_) 50 arbitrary units (a.u.), heater gas 50 a.u., curtain gas 25 a.u., collision activation dissociation, high, temperature 550 °C. Each hormone was detected using MRM transitions that were previously optimized using each standard and deuterium-labelled standard. Concentrations were determined using a standard curve prepared from a series of standard samples containing different hormone concentrations.

### Gene expression

Total RNA was extracted from *Arabidopsis* leaves finely ground in liquid nitrogen using a sterile mortar and pestle using the RNeasy Plant Mini Kit (Qiagen) according to the manufacturer’s instructions. After assessing RNA quality spectrophotometrically, genomic contamination was enzymatically degraded and verified by using a primer pair that spanned an intronic region (*AtLMCO4*, Supplementary Table S1 available at *JXB* online).

Transcript levels were measured in duplicate by quantitative real-time PCR (qRT-PCR) using Absolute Blue qPCR SYBR low ROX mix (Fisher Scientific) according to the manufacturer’s instructions. Each well contained Blue qPCR SYBR low Rox (Fisher), 1nM or 3nM forward and reverse primers, and cDNA (1/10 dilution). The following PCR program was used: 95 °C for 15min followed by 40 cycles of 95 °C for 15 s, annealing temperature for 30 s (Supplementary Table S1 at *JXB* online), 72 °C for 30 s. Dissociation curves confirmed amplicon purity. Two technical replicates were performed.

From the standard curve, relative gene expression was measured. Expression of two reference genes [*AtAct2/7* and *AtUnk* (At4g26410)] was not affected by treatment [L*er*: *AtAct2/7 F*_(2,9)_=0.73, *P*=0.51; *AtUnk F*_(2,9)_ <0.19, *P*=0.83; quad-*della* mutant: *AtAct2/7 F*_(2,9)_=2.43, *P*=0.143; *AtUnk F*_(2,9)_=0.42, *P*=0.67]. The geometric mean of *AtAct2/7* and *AtUnk* was used to normalize expression of genes of interest (Vandesompele *et al.*, 2002; Brunner *et al.*, 2004; Pfaffl *et al.*, 2004).

### Defence protein analysis

#### Protein extraction

Samples were ground to a fine powder in liquid nitrogen. Proteins were extracted in ice-cold extraction buffer, 0.1M sodium phosphate buffer, pH 7.0 containing 0.1% Triton X-100 and 7% polyvinylpyrrolidone. For the extraction of proteins to be analysed for LCMO activity, a broad-spectrum proteinase inhibitor solution (1×) was added to prevent protein degradation. Samples were vigorously vortexed and centrifuged at 15 700 *g* for 10min. Supernatants were used for protein assays.

#### Trypsin inhibitor (TI) assay

Leaf TI activity was measured according to Lara *et al.* (2000). In a 96-well plate format, trypsin (0.5 μg) was added to samples prepared in triplicate and incubated for 20min at 37 °C with gentle shaking in a Infinite M200 Pro microplate reader (Tecan). The trypsin substrate, *N*-benzoyl-dl-arginyl-β-naphthylamine (final concentration: 3mM), was added. After an 80min incubation, the reaction was inhibited by the addition of 4% HCl. After addition of the colorimetric reagent, *p*-dimethyl-amino-cinnamaldehyde (final concentration: 0.24%), the product absorbance was read at 540nm. All plates contained negative controls and a standard curve of soybean TI (concentration range, 0–1.0 μg l^–1^).

#### Laccase-like multicopper oxidase (LMCO) activity

LMCO, also known as polyphenol oxidase (PPO), activity was measured according to Espín *et al.* (1997) with minor modifications. To samples in triplicate, *N*,*N*-dimethyl formamide (final concentration: 2%), 3-methyl-2-benzothiazolinone hydrozone hydrochloride monohydrate (MBTH, final concentration: 2mM), and dopamine hydrochloride (final concentration: 35mM) are sequentially added. Controls included tyrosinase- and enzyme-free and boiled controls. Activity was monitored by measuring absorbance at 476nm at 30 s intervals for 5min at 35 °C and LMCO activity was calculated using the molar extinction coefficient of the MBTH–quinone adduct (20 700M^–1^ cm^–1^).

### Modified Bradford assay

Soluble protein concentration in leaf extracts was measured by a modified Bradford assay using a bovine serum albumin (BSA) standard curve (5–100 μg ml^–1^) (Bradford, 1976; Zor and Selinger, 1996). Samples and BSA standard curve were incubated with Bradford reagent for 10min followed by measurement of absorbance at 590nm and 450nm. The OD_590_/OD_450_ ratio was used to calculate the soluble protein concentration.

### Glucosinolate analysis

GS analysis was performed as previously described (Hogge *et al.*, 1988; Kliebenstein *et al.*, 2001). Lyophilized samples were finely ground using a pre-cooled TissueLyser (Qiagen), and 50.0mg of dry material was weighed in a 2ml Eppendorf tube and extracted twice with 1ml of 70% methanol solution, followed by 15min ultrasonification. During the first extraction, the tube was placed in a 90 °C water bath for 10min after the addition of the methanol to inhibit any myrosinase activity immediately. After sonification, tubes were centrifuged at 2975 *g* for 10min. Pooled supernatants were cleaned up by ion exchange chromatography using a diethylaminoethyl Sephadex A-25 column pre-conditioned with sterile MilliQ water. After washing with 70% methanol (2×1ml), MilliQ water (2×1ml), and 20mM sodium acetate buffer, pH 5.5 (1×1ml), GSs were desulphated by the addition of 10U of arylsulphatase and incubated at room temperature overnight. Desulphated GSs were eluted with sterile MilliQ water (2×0.75ml). The combined eluate was freeze-dried and redissolved in MilliQ water (1ml).

Desulphoglucosinolates were separated by high-performance liquid chromatography (DIONEX summit HPLC) on a reversed phase C_18_ column (Alltima C_18_, 150×4.6mm, 3 μm, Alltech) using an ACN–water gradient (2–35% ACN from 0 to 30min; flow rate 0.75ml min^–1^). Compounds were detected by a photodiode array detector (PDA). Peaks were integrated at 229nm (EC, 1990).

GSs were identified based on retention time, UV spectrum, MS analysis of selected *A. thaliana* reference samples, and the following reference standards (Phytoplan, Germany); glucoiberin (3-methylsulphenylpropylGSL), glucoerucin (4-methylthiobutylGSL), progoitrin (2-hydroxy-3-butenylGSL), sinigrin (2-propenylGSL), gluconapin (3-butenylGSL), glucobrassicanapin (4-pentenylGSL), glucobrassicin (indol-3-ylmethylGSL), sinalbin (4-hydoxybenzylGSL), glucotropaeolin (benzylGSL), and gluconasturtiin (2-phenylethylGSL). Sinigrin (63, 188, 375, 500, and 625 μM; Sigma-Aldrich) was used as an external standard. Correction factors were used to calculate GS concentrations based on the sinigrin reference curve (Buchner, 1987; EC, 1990; Brown *et al.*, 2003).

### Statistical analysis

GAs are involved in multiple aspects of plant development (Davière and Achard, 2013). Therefore, to avoid potentially confounding phenological differences between wild-type L*er* and quad-*della* mutant plants, statistical differences (*P* ≤ 0.05) were determined within each genotype by one-factor analysis of variance (ANOVA) using SPSS version 20 (SPSS Inc.) followed by a Tukey HSD post-hoc test. The results from statistical analyses are shown in Supplementary Table S2 at *JXB* online. Gene expression can be highly variable; therefore, either a statistically significant difference or a >5-fold increase over constitutive control levels was considered as an increase in transcript levels.

## Results

### Caterpillar herbivory results in a foliar labial saliva-dependent jasmonate burst

**Fig. 1.**
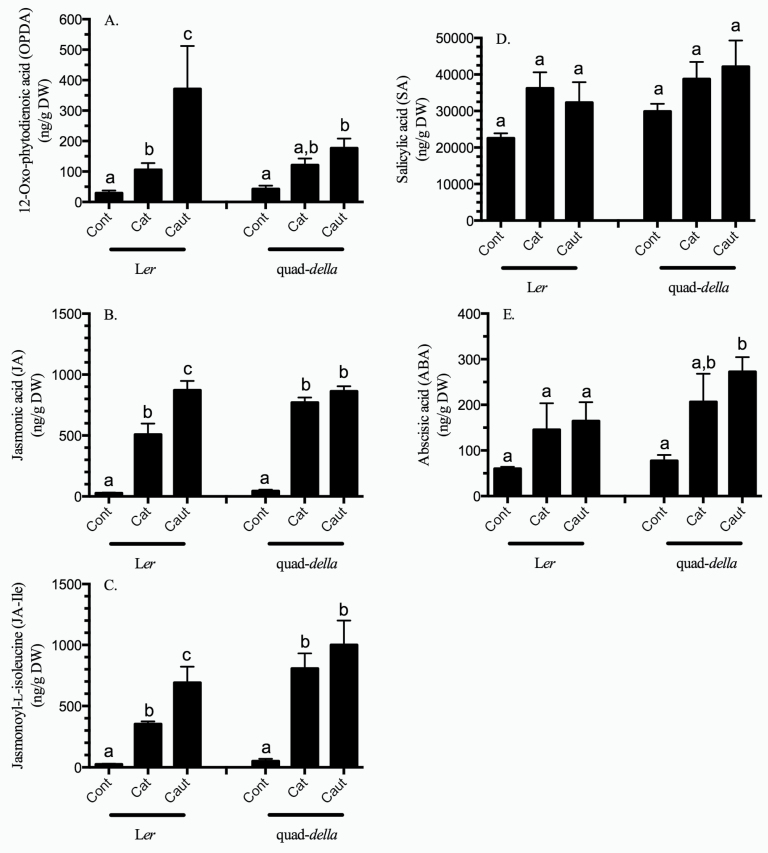
Phytohormone levels in *Arabidopsis* rosette leaves subject to caterpillar herbivory. *Arabidopsis* plants (L*er*, L*er*+GA, and quad-*della* mutant) were subject to herbivory by *Spodoptera exigua* caterpillars with intact (cat) or impaired (caut) labial salivary secretions for 10h. Plant hormones (A) 12-oxo-phytodienoic acid (OPDA), (B) jasmonic acid (JA), (C) jasmonoyl-isoleucine (JA-Ile), (D) salicylic acid (SA), and (E) abscisic acid (ABA) were measured by LC-MS/MS. Bars represent the means of 3–4 independent biological replications ±SE. Letters indicate significant differences in response to caterpillar herbivory (*P*<0.05) (Supplementary Table S2 at *JXB* online).

A rapid jasmonate (OPDA, JA, and JA-Ile) burst was observed systemically in response to caterpillar herbivory (Fig. 1A–C; Supplementary Table S2 at *JXB* online). It is important to note that significantly higher jasmonate levels were observed in plants attacked by caterpillars with impaired LS secretions compared with normal caterpillars (Fig. 1A–C; Supplementary Table S2). This LS-dependent suppression of JA-related hormone levels was alleviated in the quad-*della* mutant, indicating that DELLA proteins are required for the caterpillar LS-mediated interference with plant defence responses.

Caterpillar- or LS-dependent changes in SA hormone levels were not observed in the wild type or quad-*della* mutant (Fig. 1D; Supplementary Table S2 at *JXB* online). ABA levels were highly variable and, although a trend might be seen, further studies are needed to understand the role of ABA in these interactions (Fig. 1E; Supplemental Table S2).

### Early gene expression in response to caterpillar herbivory

**Fig. 2.**
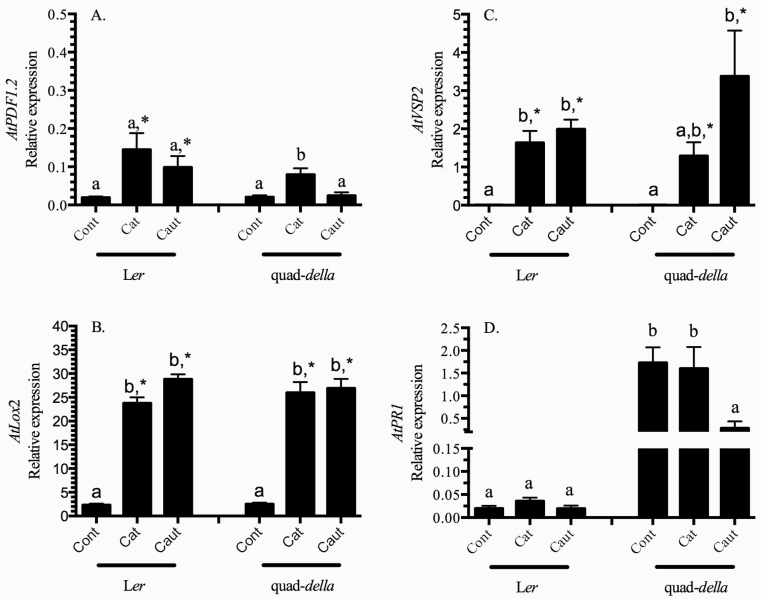
Defence gene expression in *Arabidopsis* rosette leaves in response to caterpillar herbivory. *Arabidopsis* plants (L*er*, L*er*+GA, and quad-*della* mutant) were subject to herbivory by *Spodoptera exigua* caterpillars with intact (cat) or impaired (caut) labial salivary secretions for 10h. Expression levels of marker genes of the jasmonate pathway (A) *AtPDF1.2* (JA and ethylene dependent), (B) *AtVSP2* (JA and MYC2 dependent), (C) *AtLOX2* (JA and MYC2 dependent), and (D) *PR1* (SA/NPR1 dependent) were measured by quantitative real-time PCR and normalized by the expression of two reference genes (*AtAct2/7* and *AtUnk*). Bars represent the means of 3–4 independent biological replications ±SE. Letters indicate significant differences in response to caterpillar herbivory (*P*<0.05) (Supplementary Table S2 at *JXB* online). An asterisk indicates a 5-fold increase in expression levels compared to control plants.

Early transcript expression of defence-related genes was analysed in caterpillar-wounded tissues. Expression of the JA-dependent marker gene *AtPDF1.2* increased >5-fold in response to caterpillar herbivory (Fig. 2A; Supplementary Table S2 at *JXB* online); an LS-dependent difference was not observed in wild-type plants. In comparison, in the quad-*della* mutant, an increase in *AtPDF1.2* expression was dependent upon caterpillar secretion of LS. Both *AtLOX2* and *AtVSP2* exhibited the same expression pattern and were strongly induced in response to herbivory in the wild-type L*er* and quad-*della* mutant plants (Fig. 2B, C; Supplementary Table S2); a caterpillar LS effect was not observed.

Caterpillar herbivory did not affect *AtPR1* expression in wild-type plants (Fig. 2D; Supplementary Table S2 at *JXB* online). In comparison, high constitutive *AtPR1* levels in the quad-*della* mutant plants were suppressed in response to herbivory by caterpillars with impaired LS secretions.

### Caterpillar herbivory results in an increase in the indole glucosinolate 4-methoxyglucobrassicin (4-MGB)

**Fig. 3.**
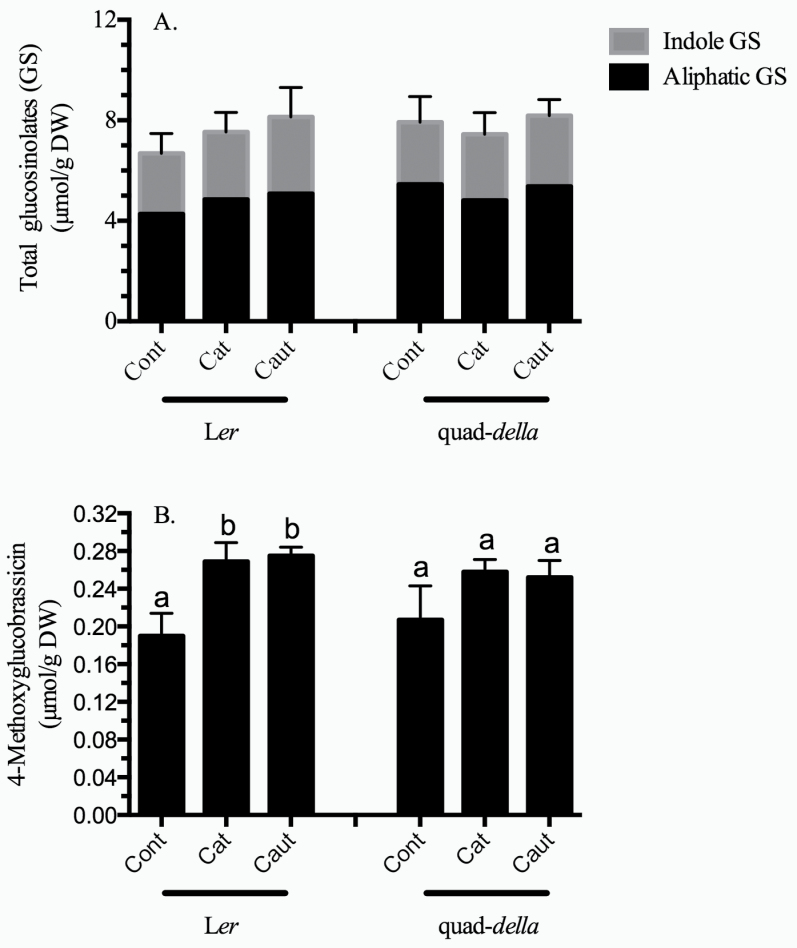
Glucosinolate (GS) profile in *Arabidopsis* rosette leaves subject to caterpillar herbivory. *Arabidopsis* plants (L*er*, L*er*+GA, and quad-*della* mutant) were subject to herbivory by *Spodoptera exigua* caterpillars with intact (cat) or impaired (caut) labial salivary secretions for 10h. Compounds extracted from lyophilized samples were desulphated and subjected to HPLC analysis. (A) Total GS profile in *Arabidopsis* plants; a significant change in total or individual GS levels was not observed under these treatments, with the exception of 4-methoxyglucobrassicin (4-MGB) which is highlighted in (B). Bars represent the means of 3–4 independent biological replications ±SE. Letters indicate significant differences in response to caterpillar herbivory (*P*<0.05) (Supplementary Table S2 at *JXB* online).

Local defence responses of the plant were measured through the analysis of secondary metabolites and defence-related proteins. Both indole and aliphatic GSs were identified in L*er* leaves (Table 1, Fig. 3A). Though indole GS levels were comparable with previous reports, lower levels of aliphatic compounds were identified in this study, which may reflect the differences in growth conditions (Kliebenstein *et al.*, 2001; Brown *et al.*, 2003); an ~50% decrease in levels of the main aliphatic GS, 2-hydroxypropyl GS, accounts for much of this discrepancy.

**Table 1.** *Glucosinolate (GS) levels in* Arabidopsis *rosette leaves subject to caterpillar herbivory*Five-week-old *Arabidopsis thaliana* subject to herbivory by fourth instar *Spodoptera exigua* caterpillars for 10h (*n*=4). Caterpillars had either intact (cat.) or impaired (caut.) labial salivary secretions.

GS (nmol g^–1^ DW)	L*er*			quad-*della* mutant		
	Control	Cat.	Caut.	Control	Cat.	Caut.
3-Hydroxypropyl GS	4020.7±516.4	4415.8±604.4	4792.3±897.1	4966.4±712.4	3820.9±674.8	4850.7±499.8
Glucoiberin	196.0±84.5	258.9±113.4	182.3±5.9	331.0±109.4	296.9±83.1	381.1±118.2
Glucoraphanin	93.1±11.43	233.0±138.3	112.5±21.5	142.4±24.0	85.8±9.4	126.5±16.4
Glucobrassicin	2203.4±293.9	2392.3±297.8	2738.6±300.7	2251.7±256.6	2335.3±230.5	2534.2±89.2
Neo-glucobrassicin	29.9±6.0	39.7±8.3	47.5±8.0	25.5±5.1	35.3±3.8	29.4±3.4
4-Methyoxyglucobrassin	190.3±24.3 a	269.4±20.1 b	275.1±9.8 b	207.0±36.1 a	258.4±13.1 a	252.1±18.4 a

A significant increase in the GS 4-methoxyglucobrassicin was observed in response to herbivory in wild-type *Arabidopsis* [L*er*: *F*_(2,9)_=6.17, *P*=0.02].

Letters indicate significant differences due to herbivory within each genotype (L*er* or quad-*della* mutant).

Levels of aliphatic GSs were not affected by caterpillar herbivory (Table 1; Supplementary Table S2 at *JXB* online). In contrast, 4-MGB was induced ~25–40% in response to caterpillar herbivory in L*er* but not in the quad-*della* mutants (Fig. 3A, B, Table 1; Supplementary Table S2). Levels of the other indole GSs did not change upon caterpillar feeding.

An LS-specific induction of GS levels was not observed (Fig 3A, B, Table 1; Supplementary Table S2 at *JXB* online). However, the increase in 4-MGB observed in response to caterpillar herbivory was alleviated in the quad-*della* mutant, suggesting that DELLA proteins are important in the JA-dependent regulation of GS biosynthesis.

### Caterpillar herbivory does not affect early defensive protein activity: TI and LMCO

**Fig. 4.**
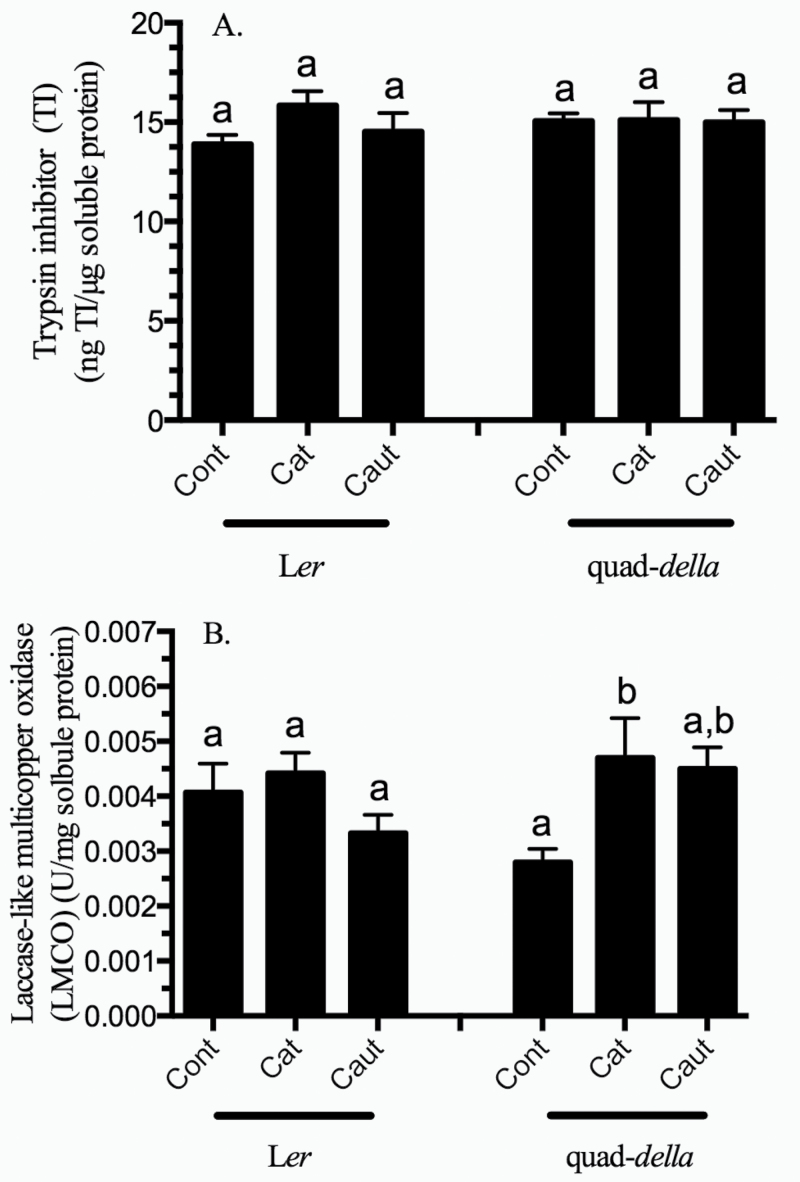
Levels and activities of defensive proteins in *Arabidopsis* rosette leaves subject to caterpillar herbivory. Five-week-old *Arabidopsis* plants (L*er*, L*er*+GA, and quad-*della* mutant) were subject to herbivory by fourth instar *Spodoptera exigua* caterpillars with intact (cat) or impaired (caut) labial salivary secretions for 10h. Defensive proteins, (A) trypsin inhibitor levels and (B) laccase-like multicopper oxidase (LMCO) activity, were measured. Bars represent the means of 3–4 independent biological replications ±SA. Letters indicate significant differences in response to caterpillar herbivory (*P*<0.05) (Supplementary Table S2 at *JXB* online).

Constitutive TI levels did not increase in the early response to caterpillar herbivory or LS in either wild-type L*er* or the quad-*della* mutant plants (Fig. 4A; Supplementary Table S2 at *JXB* online). In wild-type L*er* plants, constitutive LMCO activity did not increase in response to herbivory (Fig. 4B; Supplementary Table S2). In comparison, a significant increase in LMCO activity was observed in the quad-*della* mutant when plants were infested by caterpillars with intact salivary secretions.

## Discussion

### Responses to caterpillar herbivory

As a plant faces multiple challenges in the environment, there are trade-offs between growth and defence. Two key hormone systems that regulate these physiological processes are GA/DELLA proteins for growth and JAs/JAZ proteins involved in plant defence against chewing herbivores, such as caterpillars (Ballare, 2011; Robert-Seilaniantz *et al.*, 2011). The cross-talk between these two pathways integrates environmental information with plant development to shape the physiological response of the plant. JA interferes with the GA-mediated degradation of the negative growth regulator DELLA proteins (Heinrich *et al.*, 2012; Yang *et al.*, 2012). In addition, DELLA proteins enhance JA-dependent responses by competing with the transcriptional activator MYC2 for the negative regulator JAZ proteins (Hou *et al.*, 2010; Wild *et al.*, 2012). This study investigated the potential cross-talk between the GA/DELLA and the JA pathway in the early plant responses to caterpillar herbivory (10h). In addition, the role of caterpillar LS effector(s) in these interactions was determined.

Caterpillar infestation of both wild-type and the quad-*della* mutant plants results in a strong systemic jasmonate burst, as has been witnessed in many other plant–caterpillar models, including wild tobacco–*Manduca sexta* and tomato–*Helicoverpa zea* (Fig. 1A–C) (Diezel *et al.*, 2009; Tian *et al.*, 2012). In contrast, caterpillar-specific changes in SA hormone levels are not observed in these two genotypes, as was also noted by Weech *et al.* (2008) and Tian *et al.* (2012) (Fig. 1D).

Transcript expression of marker genes of the JA and SA pathways was further analysed. *AtVSP2* and *AtLOX2* are well characterized markers of the MYC2 branch of the JA pathway (Bell and Mullet, 1993; Dombrecht *et al.*, 2007; Kazan and Manners, 2013). *AtPDF1.2* is induced synergistically in response to JA and ethylene, negatively regulated by MYC2, and late expression requires the NPR1/TGA pathway (Penninckx *et al.*, 1998; Zander *et al.*, 2010). Given the strong jasmonate burst, it is not surprising that in L*er* wild-type and quad-*della* mutant plants, *AtVSP2*, *AtLOX2*, and *AtPDF1.2* are strongly induced in response to caterpillar herbivory (Fig. 2A–C).

In contrast, caterpillar herbivory did not affect SA hormone levels or expression of the SA-dependent gene *AtPR1* (Figs 1D, 2D). Tian *et al.* (2012) also found that SA-dependent, early gene expression was not affected by caterpillar herbivory. In stark contrast, Paudel *et al.* (2013) observed a strong 5-fold induction of *AtPR1* expression in response to caterpillar herbivory. This probably reflects temporal differences in the experimental design where in this study and that of Tian *et al.* (2012) gene expression was evaluated at ≤10h after the initiation of herbivory compared with that of Paudel *et al.* (2013) where *AtPR1* transcript levels were measured 36h after herbivory.

GSs are the principal defensive compound in *Arabidopsis* (Halikier and Gershenzon, 2006; Hopkins *et al.*, 2009). Levels of aliphatic GSs are not affected by caterpillar herbivory (Table 1), in contrast to previous studies where, in the Col background, Mewis *et al.* (2005) noticed an increase in short-chain aliphatic methylsulphinyl GS in response to *S. exigua* herbivory. However, the levels and types of GS and, presumably, the regulation differ between *Arabidopsis* genotypes (Kliebenstein *et al.*, 2001; Kusnierczyk *et al.*, 2007). In response to caterpillar feeding, local levels of the indole GS 4-MGB significantly increase (Fig. 3A). Principal component analysis of *Arabidopsis* ecotypes identified this GS as an important compound negatively effecting *S. exigua* larval growth (Mosleh Arany *et al.*, 2008). However, this increase in 4-MGB was only observed in wild-type but not in the quad-*della* mutant plants, suggesting that DELLA proteins may be involved in the regulation of some branches of GS biosynthesis.

TI or LMCO activity do not increase in the early responses of wild-type *Arabidopsis* plants to caterpillar herbivory (Fig. 4A, B). In comparison, LMCO increases in quad-*della* mutant plants infested by caterpillars with intact salivary secretions. This result was unexpected. However, LMCO enzymes are involved in many physiological functions in the plant, including the lignification of cell walls (Thipyapong *et al.*, 1997; Cai *et al.*, 2006; Constabel and Barbehenn, 2008). Therefore, DELLA proteins may negatively regulate LMCO activity in response to caterpillar herbivory.

Together, these data support previous research which shows that in response to stress, JA-mediated defence responses take priority over GA-dependent growth processes (Hou *et al.* 2010; Heinrich *et al.*, 2012; Wild *et al.*, 2012; Yang *et al.*, 2012). The present data suggest that DELLA proteins may be involved in the regulation of GSs and also suppress LMCO activity, which may be related to their role in plant cell wall fortification (Thipyapong *et al.*, 1997; Cai *et al.*, 2006; Constabel and Barbehenn, 2008).

### Caterpillar labial saliva-specific responses

Since caterpillar LS has been implicated as a stratagem to modify plant-induced defences (Musser *et al.*, 2002; Weech *et al.*, 2008; Tian *et al.*, 2012), plant-induced responses to caterpillars with intact versus impaired LS secretions were compared. *Arabidopsis* plants subject to herbivory by caterpillars with impaired LS secretions have significantly higher jasmonate levels (OPDA, JA, and JA-Ile) compared with normal *S. exigua*, indicating that the LS contains effector(s) that suppress this jasmonate burst in response to herbivory (Fig. 1A–C). Weech *et al.* (2008) observed a similar distinction in JA levels between plants infested by caterpillars with intact and impaired salivary secretions. In contrast, in the quad-*della* mutants, the LS-dependent difference in jasmonate levels is not observed (Fig. 1A–C). Therefore, DELLA proteins are required for caterpillar LS-dependent suppression of plant defence hormones.

Even though an LS-specific difference in jasmonate levels is observed, the expression of JA-dependent genes shows a slightly different pattern (Fig. 2A–C). Expression of *AtPDF1.2*, *AtLOX2*, and *AtVSP2* is strongly induced in response to herbivory; however, caterpillar LS differences in transcript expression are not observed. Similar observations for *AtLOX2* have been made previously (Weech *et al.*, 2008; Tian *et al.*, 2012; Paudel *et al.*, 2013). However, *AtPDF1.2* suppression by caterpillar LS effectors is well recognized (Weech *et al.*, 2008; Paudel *et al.*, 2013). This probably reflects the temporal regulation of this gene. Zander *et al.* (2010) have shown that the SA/NPR1-dependent TGA transcription factors regulate late but not early *AtPDF1.2* gene epression, and caterpillar LS-mediated suppression of plant induced defences is believed to involve the SA/NPR1/TGA pathway possibly by a mechanism as elucidated by Van der Does *et al.* (2013) (Weech *et al.*, 2008; Paudel *et al.*, 2013).

In the quad-*della* mutant, expression of *AtLOX2* and *AtVSP2* parallels that of wild-type plants (Fig. 2B, C). In contrast, expression of *AtPDF1.2* was only induced in response to herbivory by caterpillars with intact salivary secretions in the quad-*della* mutant, suggesting a complex relationship with DELLA proteins in the regulation of this gene (Fig. 2A).

A caterpillar LS-specific difference in SA levels was not observed, and this is reflected in the expression of the marker gene *AtPR1* in the wild-type plant (Figs 1D, 2D). In contrast, high constitutive *AtPR1* levels of the quad-*della* mutant were suppressed in response to herbivory by caterpillars with impaired LS secretions (Fig. 2D). A possible explanation is that herbivory by caterpillars with impaired LS secretions leads to a strong activation of JA responses which is known to interfere with the SA/NPR1 pathway and, thus, a suppression of *AtPR1* expression is observed (Laurie-Berry *et al.*, 2006; Zarate *et al.*, 2007).

Plant defensive compounds and protein activity analysed in this study were not affected by caterpillar LS (Fig. 3 and 4).

### Conclusion

**Fig. 5.**
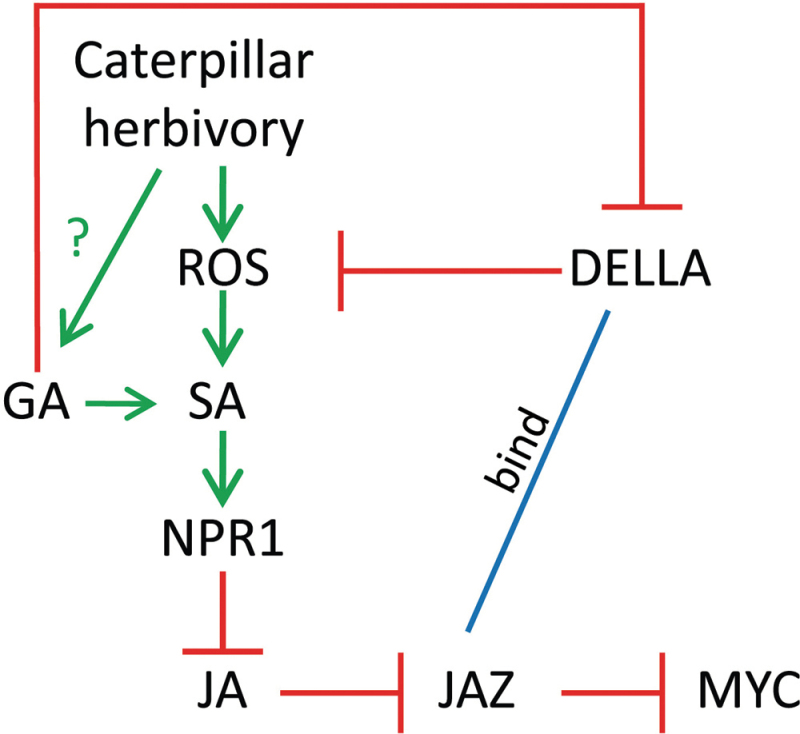
Proposed model of the gibberellin/DELLA pathway in plant–insect interactions. Caterpillar labial salivary secretions delay or suppress induced plant defences. The underlying mechanism involves cross-talk between plant signalling pathways. DELLA proteins are involved in the labial saliva-dependent suppression of the jasmonate burst. In response to caterpillar herbivory, DELLA proteins suppress laccase-like multicopper oxidase activity and positively regulate glucosinolate biosynthesis. Arrows indicate positive effects, bar-headed lines represent inhibitory effects, and dashed lines indicate putative interactions. GA, gibberellin; JA, jasmonates; JAZ, jasmonate ZIM-domain proteins; NPR1, nonexpressor of pathogenesis-related protein 1; ROS, reactive oxygen species; SA, salicylic acid. (This figure is available in colour at *JXB* online.)

The present results show a link between DELLA proteins and the regulation of plant defences, such as GSs, in response to insect stress (Fig. 4B) and in the caterpillar LS-mediated suppression of plant defence hormone biosynthesis (Fig. 1A–C). Previous models propose that caterpillar LS effector(s) manipulate plant defences through the generation of ROS, such as hydrogen peroxide, that activate the NPR1/TGA pathway to modulate induced plant defences (Eichenseer *et al.*, 1999; Musser *et al.*, 2002; Weech *et al.*, 2008; Tian *et al.*, 2012; Paudel *et al.*, 2013). DELLA proteins are known to scavenge hydrogen peroxide (Achard *et al.*, 2008). In addition, treatment of *Arabidopsis* with GAs results in the activation of the NPR1 pathway (Alonso-Ramírez *et al.*, 2009). Therefore, in the quad-*della* mutant, a stronger LS-dependent response was expected, but was not observed. The mechanism underlying the involvement of GA/DELLA in these plant–insect interactions is as yet unknown but may involve competition between DELLA proteins and MYC transcription factors for negative regulator JAZ proteins (Hou *et al.*, 2010; Wild *et al.*, 2012; Yang *et al.*, 2012). Therefore, there appear to be multiple points of cross-talk between the JA defence pathway and the GA/DELLA pathway to ensure prioritization of plant responses to changing environmental conditions (Fig. 5). Future studies will continue to further elucidate the underlying mechanism.

## Supplementary data

Supplementary data are available at *JXB* online.

Table S1. Primers to check for genomic contamination and quantitative real-time PCR.

Table S2. Statistical results of plant–insect experiments.

Supplementary Data

## Abbreviations:

ABA: abscisic acid
Cat: caterpillars with intact labial salivary secretions
Caut: caterpillars with impaired labial salivary secretions
GA: gibberellin
GOX: glucose oxidase
GS: glucosinolate
JA: jasmonic acid
JA-Ile: 7-jasmonoyl-l-isoleucine
JAZ: jasmonate ZIM-domain
LMCO: laccase-like multicopper oxidase
LOX2: lipoxygenase 2
LS: labial saliva
NPR1: nonexpressor of pathogenesis-related protein 1
OPDA: 12-oxo-phytodienoic acid
PDF1.2: plant defensin 1.2
PR1: pathogenesis-related 1
ROS: reactive oxygen species
SA: salicylic acid
TI: trypsin inhibitor
VSP2: vegetative storage protein 2.
