# Supplementary material for: DELLA proteins modulate Arabidopsis defences induced in response to caterpillar herbivory
Source: J Exp Bot. 2014 Jan 7;65(2):571–83. doi: 10.1093/jxb/ert420 (PMC3904718; doi:10.1093/jxb/ert420)
Supplement: Supplementary Data [file supp_65_2_571__index.html]

DELLA proteins modulate Arabidopsis defences induced in response to caterpillar herbivory — DELLA proteins modulate Arabidopsis defences induced in response to caterpillar herbivory — Supplementary Data 

# DELLA proteins modulate *Arabidopsis* defences induced in response to caterpillar herbivory

## Supplementary Data

Data files

**Files in this Data Supplement:**

- Supplementary Data - Supplementary Data
