# Supplementary material for: DELLA proteins modulate Arabidopsis defences induced in response to caterpillar herbivory
Source: J Exp Bot. 2014 Jan 7;65(2):571–83. doi: 10.1093/jxb/ert420 (PMC3904718; doi:10.1093/jxb/ert420)
Supplement: Supplementary Data [file supp_ert420_jexbot106682_file001.pdf]

Supplemental Table 1. Primers to check for genomic contamination and quantitative real time-polymerase chain reaction (qRT-PCR).

Gene	Accession number	Annealing temperature (°C)	Forward (5’-3’)	Reverse (5’-3)	Ref.
AtLMCO4	NM_129364		ATG GGT CGT CAT CAG ATT CAG AGC AGA TAA	CAT ATA AGA GGT GTG TTA GAG ACA ATA ATA	**Weech *et al.* (2008)**
AtACT2/7	At5g09810	60	GTA TGC TCT TCC TCA TGC TAT CCT T	TTC CCG TTC TGC GGT AGT G	**Beste *et al.* (2011)**
AtUnk	At4g26410	60	GAG CTG AAG TGG CTT CCA TGA C	GGT CCG ACA TAC CCA TGA TCC	**Czechowski et al. (2005)**
AtPDF1.2b	At2g26020	59	CGG CAA TGG TGG AAG CA	CAT GCA TTA CTG TTT CCG CAA	**Jirage *et al.* (2001)**
AtLOX2	At3g45410	57	GTC CTA CTT GCC TTC CCA AAC	ATT GTC AGG GTC ACC AAC ATC	**Weech *et al.* (2008)**
AtVSP2	At5g24770	58	ATA TGG ATA CGG AAC AGA GAA G	CCA TTA GGC TTC AAT ATG AGA T	**Troufflard *et al.* (2010)**
AtPR1	At2g14610	58	CAC TAC ACT CAA GTT GTT TGG A	TAG TAT GGC TTC TCG TTC ACA	**Kuśnierczyk *et al.* (2007)**

Supplemental Table 2. Statistical results of plant-insect experiments. Significant differences were analyzed within each plant genotype. A one-factor analysis of variance (ANOVA) followed by a Tukey HSD was used to compare caterpillar treatments within each genotype (L*er* and quad-*della* mutant)

	**L*er* wildtype**		**quad-*della* mutant**	
	F value	P value	F value	P value
**12-Oxo-phytodienoic acid (OPDA)**	$F_{(2, 9)} = 4.74$	$p = 0.04$	$F_{(2, 9)} = 8.70$	$p = 0.01$
**Jasmonic acid (JA)**	$F_{(2,8)} = 38.28$	$p < 0.001$	$F_{(2, 9)} = 169.14$	$p < 0.001$
**Jasmonoyl-L-isoleucine (JA-Ile)**	$F_{(2,9)} = 18.72$	$p = 0.001$	$F_{(2, 9)} = 13.50$	$p = 0.002$
**Salicylic acid (SA)**	$F_{(2, 9)} = 2.84$	$p = 0.11$	$F_{(2, 9)} = 1.55$	$p = 0.26$
**Abscisic acid (ABA)**	$F_{(2, 9)} = 1.82$	$p = 0.22$	$F_{(2, 9)} = 5.93$	$p = 0.02$
AtPDF1.2	$F_{(2, 8)} = 3.31$	$p = 0.09$	$F_{(2, 9)} = 8.54$	$p = 0.01$
AtLOX2	$F_{(2, 9)} = 212.16$	$p < 0.001$	$F_{(2, 9)} = 63.53$	$p < 0.001$
AtVSP2	$F_{(2, 8)} = 13.57$	$p = 0.003$	$F_{(2, 9)} = 5.54$	$p = 0.03$
AtPR1	$F_{(2, 8)} = 1.82$	$p = 0.22$	$F_{(2, 9)} = 4.933$	$p = 0.046$
AtUnk	$F_{(2, 9)} = 0.19$	$p = 0.83$	$F_{(2, 9)} = 0.42$	$p = 0.67$
AtAct2	$F_{(2, 9)} = 0.73$	$p = 0.51$	$F_{(2, 9)} = 2.43$	$p = 0.14$
**Trypsin inhibitor (TI)**	$F_{(2, 21)} = 0.57$	$p = 0.58$	$F_{(2, 20)} = 0.01$	$p = 0.99$
**Laccase-like multicopper oxidase (LMCO)**	$F_{(2, 21)} = 1.75$	$p = 0.20$	$F_{(2, 21)} = 4.57$	$p = 0.02$
